# Supplementary material for: Fake news, misinformation, vaccine hesitancy and the role of community engagement in COVID-19 vaccine acceptance in Southern Ghana
Source: PLoS One. 2025 Jun 10;20(6):e0316969. doi: 10.1371/journal.pone.0316969 (PMC12151338; doi:10.1371/journal.pone.0316969)
Supplement: Attachment — Submitted filename: Comments FAKE NEWS.pdf [file pone.0316969.s001.pdf]

Comments

Title: “People will not even bring out their children to be immunised, because of the corona vaccine”: fake news, misinformation, vaccine hesitancy and the role of community engagement in COVID-19 vaccine acceptance in Southern Ghana

ABSTRACT

Introduction: Emphatic statement like… *According to the World Health Organisation* should be reconsidered since there are no in-text citations

Method: There is nothing like qualitative case study. The authors should consider the the use of the term appropriately as case study. In-depth interviews is a procedure and NOT A TOOL. THEREFORE, the use of in-depth interviews in that context of the study is appropriate in the method section of the abstract

The use of community members should be specified since the data were collected from specified participants in the community

Conclusion: This appears more of recommendations and not conclusion. Again, if they are recommendations, they are generally sweeping and should be specified

METHOD

Study design

It will be appropriate to discuss only the study approach here. And discuss the data collection procedure subsequently together with the role of each member. Fusing all together with a heading as study design in my view is problematic.

STUDY SETTING

Is Volta region considered as Southern Ghana? Again, this needs to be justified properly.

2.4. Training of data collectors and quality control

In line 199, the use of validity and reliability is **NOT correct** SINCE qualitative research cannot establish that in the data collection tool. The authors need to actually explain the rigor employed in the study.

RESULT

This section is detailed enough

However, under line 523, the use of the heading **Impact of limited engagement** in addressing misinformation is problematic since qualitative study cannot establish **impact.** Try to rephrase it.

5.0 Recommendation and conclusion

Conclusion should rather come before recommendations
