# Supplementary material for: Fake news, misinformation, vaccine hesitancy and the role of community engagement in COVID-19 vaccine acceptance in Southern Ghana
Source: PLoS One. 2025 Jun 10;20(6):e0316969. doi: 10.1371/journal.pone.0316969 (PMC12151338; doi:10.1371/journal.pone.0316969)
Supplement: Attachment — Submitted filename: Response to Reviewers comments.docx [file pone.0316969.s003.docx]

**Response to reviewers’ comments**

**Reviewer #1**

Thank you for your careful review, which would go a long way towards improving the quality of the paper. Kindly find below the responses to the issues raised.

**Reviewer’s Comment:** The title stresses on childhood vaccination. I am not sure why this is the case. The later part after the quotation marks in the title would suffice.

**Response:** The title has been revised accordingly, **kindly see lines 1 and 3**

**Reviewer’s Comment:** Again in the discussion, the details of impact of childhood vaccination were discussed in much details and that was not reflected in the results that much.

**Response:** The discussion point on childhood vaccination in this current study is based on the results that parents lost trust in health workers as they were afraid that due to their refusal to vaccinate against covid-19, health workers were using the childhood vaccination as an excuse to give their children covid-19 vaccines. **Kindly refer to lines 352 to 364.**

**Reviewer** # **2**

Thank you for your comments. Kindly find below responses to your comments.

**Reviewer’s Comment:** This study is unsuitable for publication in this journal because it is not scientific. Whether this is a fake disease or not should have been discussed in 2020 and 2021. It is better published in a newspaper than in a scientific journal.

**Response:** This study is scientific and suitable for publication in this esteemed journal, because it has utilized systematic and rigorous methods to explore and understand a phenomena as recommended in qualitative studies (Forero et al., 2018, Sale and Thielke, 2018). Importantly, it contributes to health care policy and decision making. Qualitative research focuses on understanding the meanings and experiences of individuals using data collection methods such as in-depth interviews, participant observations, and focus group discussions. These methods generate textual data, which require the use of qualitative data analysis methods such as NVivo. A qualitative software to support coding and analysis. Consequently, this study does not require statistical analysis to make it scientific and relevant to health policy. Additionally, this study adheres to the Consolidated Criteria for Reporting Qualitative Research (COREQ) Checklist (Tong et al., 2007), a recognized scientific and systematic reporting guideline for qualitative research, ensuring methodological rigor and credibility.

COVID-19 was and remains a relevant global public health problem, with many countries still grappling with its effects and seeking effective policy interventions. Ghana has integrated COVID-19 vaccination into routine public health care and thus, it is important that studies of this nature are conducted to inform public health policy. Additionally, the lessons from this study are relevant to other contexts and any other pandemic in the future.

**Reviewer #3**

Thank you for your careful review, which would go a long way towards improving the quality of the paper. Kindly find below the responses to the issues raised.

**ABSTRACT**
**Reviewer’s Comment:** Introduction: Emphatic statement like… According to the World Health Organisation should be reconsidered since there are no in-text citations

**Response:** The statement 'According to the World Health Organisation' has been removed to align with the absence of an in-text citation. Kindly refer to lines 28 and 29

METHOD:

**Reviewer’s Comment:** There is nothing like qualitative case study. The authors should consider the use of the term appropriately as case study.

**Response:** Thank you for the correction. Qualitative has been removed to reflect the design, **kindly refer to lines 34, 128 and 622**

**Reviewer’s Comment:** In-depth interviews is a procedure and NOT A TOOL. THEREFORE, the use of in-depth interviews in that context of the study is appropriate in the method section of the abstract

**Response:** Well noted with thanks

**Reviewer’s Comment:** The use of community members should be specified since the data were collected from specified participants in the community

**Response:** Community groups specified, **kindly see lines 38**

**Reviewer’s Comment:** Conclusion: This appears more of recommendations and not conclusion. Again, if they are recommendations, they are generally sweeping and should be specified

**Response:** It is a conclusion. It has been duly revised. **Kindly refer to lines 719 - 756**

METHOD
**Reviewer’s Comment:** Study design
It will be appropriate to discuss only the study approach here. And discuss the data collection procedure subsequently together with the role of each member. Fusing all together with a heading as study design in my view is problematic.

**Response:** The study approach and data collection procedure have been separated accordingly. **Kindly refer to lines 127 – 131 concerning the research approach and 185 - 200 for data collection procedure.**

STUDY SETTING
**Reviewer’s Comment:** Is Volta region considered as Southern Ghana? Again, this needs to be justified properly.

**Response:** Yes, the Volta Region is considered part of Southern Ghana, specifically the southeastern part (Ghana Birth and Deaths Registry, 2025). Geographically, it extends from the Gulf of Guinea in the south, where it has coastal districts, to the northern areas bordering the recently created Oti Region (Nyatuame et al., 2014).
**Reviewer’s Comment:** 2.4. Training of data collectors and quality control
In line 199, the use of validity and reliability is NOT correct SINCE qualitative research cannot establish that in the data collection tool. The authors need to actually explain the rigor employed in the study.

**Response:** Authors have explained how rigor was maintained**. Kindly refer to lines 212 to** **219.**

**RESULT**
**Reviewer’s Comment:** This section is detailed enough
However, under line 523, the use of the heading Impact of limited engagement in addressing misinformation is problematic since qualitative study cannot establish impact. Try to rephrase it.

**Response:** The heading has been changed to “Consequences of limited engagement in addressing misinformation on decision to vaccinate”. **Kindly refer to line 524**

**Reviewer’s Comment:** 5.0 Recommendation and conclusion
Conclusion should rather come before recommendations

**Response:** We have revised the section by placing the conclusion section before the recommendations**. Kindly refer to lines 722-734 for the conclusion.**

**Please refer to lines 735– 756 for the recommendations**

**BIBLIOGRAPHY**

Ghana Birth and Deaths Registry., 2025 <https://bdr.gov.gh/volta/> accessed 30/04/2025

Nyatuame, M., Owusu-Gyimah, V., & Ampiaw, F. (2014). Statistical Analysis of Rainfall Trend for Volta Region in Ghana. *International Journal of Atmospheric Sciences*, *2014*, 1–11. https://doi.org/10.1155/2014/203245

FORERO, ROBERTO, NAHIDI, S., DE COSTA, J., MOHSIN, M., FITZGERALD, G., GIBSON, N., MCCARTHY, S. & ABOAGYE-SARFO, P. 2018. Application of four-dimension criteria to assess rigour of qualitative research in emergency medicine. *BMC Health Services Research,* 18.

SALE, J. E. M. & THIELKE, S. 2018. Qualitative research is a fundamental scientific process. *Journal of Clinical Epidemiology,* 102**,** 129-133.

TONG, A., SAINSBURY, P. & CRAIG, J. 2007. Consolidated criteria for reporting qualitative research (COREQ): a 32-item checklist for interviews and focus groups. *International Journal for Quality in Health Care,* 19**,** 349-357.
